# Salivary alkaline phosphatase levels and alveolar bone crest changes in immediate vs. delayed implant placement

**DOI:** 10.34172/japid.2025.006

**Published:** 2025-02-12

**Authors:** Bahareh Hekmat, Masoumeh Amani, Farhad Aghmasheh, Zahra Mohammad Hasani

**Affiliations:** ^1^Department of Oral and Maxillofacial Radiology, School of Dentistry, Zanjan University of Medical Sciences, Zanjan, Iran; ^2^Department of Oral and Maxillofacial Surgery, School of Dentistry, Zanjan University of Medical Sciences, Zanjan, Iran

**Keywords:** Alkaline phosphatase, Alveolar bone, Bone metabolism, Immediate implant, Salivary biomarkers

## Abstract

**Background.:**

Dental implant therapy is a top option for replacing lost or unsalvageable teeth. The most common placement protocols are immediate and delayed. A key factor for successful treatment is the stability of the marginal bone, which can be evaluated through radiography and salivary biomarkers. This study evaluated the relationship between salivary alkaline phosphatase levels and the alveolar crest bone height in immediate and delayed implants.

**Methods.:**

This study involved 62 patients: 31 for immediate and 31 for delayed implant placement in posterior jaw regions. Unstimulated saliva (5 mL) was collected from all the patients before surgery and at 14- and 4-month postoperative intervals to assess salivary alkaline phosphatase activity using spectrophotometry. Intraoral periapical digital radiographs were taken immediately after surgery and 2 and 4 months postoperatively to evaluate alveolar crest bone height. Measurements at the mesial and distal implant sites were analyzed using Scanora software.

**Results.:**

No significant differences were found in salivary alkaline phosphatase activity or alveolar crest bone resorption based on gender, implant timing, or jaw region (*P*>0.05). However, patient age significantly correlated with alkaline phosphatase activity and bone resorption (*P*<0.05). There was no correlation between alkaline phosphatase levels and alveolar crest bone height.

**Conclusion.:**

Salivary alkaline phosphatase cannot be considered a reliable diagnostic biomarker for evaluating the condition of the alveolar crest bone around dental implants.

## Introduction

 Throughout dental history, addressing issues emanating from tooth loss has been essential. Today, a key goal in dental treatments is to restore function, comfort, and aesthetics and replace missing teeth through various methods.^1,2^ One of the advanced and widely accepted methods for replacing lost teeth and restoring oral function and aesthetics is implant therapy.^3^

 Various timing protocols for implant placement after tooth extraction have been discussed in the literature, including immediate implant placement after tooth extraction, early implant placement with soft tissue healing (4‒8 weeks after extraction), early implant placement with partial bone healing (12-16 weeks after extraction), and late implant placement more than 16 weeks after extraction.^4,5^

 Saliva is a fluid secreted by the major and minor salivary glands and contains various biomarkers. Biomarkers are measurable indicators used to assess biological conditions. The use of salivary biomarkers as a diagnostic tool has many advantages. Since saliva collection is easy and non-invasive, it can be used for the early diagnosis of various medical conditions, such as malignancies, metabolic diseases, infections, inflammation, and autoimmune diseases, by monitoring changes in biomarkers.^6–8^

 Alkaline phosphatase (ALP) is a biomarker of bone metabolism, with its primary sources being bone, liver, kidneys, intestines, and the placenta. It is also found in many periodontal cells, such as osteoblasts, neutrophils, and fibroblasts. ALP is released from osteoblasts for bone formation, from fibroblasts in the periodontal ligament for the repair of the periodontium, and from polymorphonuclear neutrophils during inflammation.^7,9^

 Today, one of the main criteria for the success of implant treatment is the preservation of the interdental papilla, which is influenced by the resorption of the alveolar bone crest and may jeopardize the success of the treatment. The alveolar bone crest and papilla support the periodontium adjacent to the implant, acting as a biological barrier against external factors and preventing food impaction.^10^

 Salivary ALP is one of the markers of bone regeneration, useful for assessing the bone healing process and potentially influencing the prognosis of implant treatment.^10,11^ Early implant failure has various causes, including surgical trauma, flap design, excessive occlusal forces, and infection of the tissues surrounding the implant.^10^ The pattern of salivary ALP changes mirrors those in serum, reflecting bone remodeling conditions. Increased salivary ALP levels (originating from neutrophils) indicate inflammation and destruction of healthy tissues, making it a practical and reliable biomarker.^7^

 The extent of alveolar bone crest resorption can be evaluated by assessing salivary ALP levels and reviewing patient radiographs after implant placement.^10,11^ Various studies have investigated the relationship between salivary ALP levels and implant timing, particularly focusing on how ALP levels fluctuate with immediate versus delayed implant placements. Some studies, like Fathima and Harish, suggest that ALP levels may be a reliable biomarker for inflammation and bone remodeling processes. However, the literature reports inconsistencies. For instance, while Alshibib and Saleh propose a strong positive correlation between salivary ALP and bone regeneration, other studies, such as those by Di Lenardo et al and Prakash et al, indicate weak or no significant correlation.^7,8,12^

 These discrepancies may arise from variations in study designs, sample sizes, or timing intervals used to measure ALP. The current study aims to explore this relationship further by examining salivary ALP levels across multiple time points and comparing the findings with previous literature to clarify these inconsistencies.

## Methods

 This study was conducted after receiving ethical approval (IR.ZUMS.REC.1401.057) from Zanjan University of Medical Sciences on patients visiting the Department of Oral and Maxillofacial Surgery at the Dental School of Zanjan University of Medical Sciences. The patients were candidates for immediate and delayed implants in the posterior regions of their jaws. The patients were selected based on the following criteria: no surgeries in the past three months, no systemic diseases, non-smokers, not pregnant or breastfeeding, no acute or chronic periodontitis, and no infection around the implant placement site.

 Sixty-two patients were included in the study, divided into two groups of 31:

- Group A: Patients who were candidates for immediate implants (immediately after tooth extraction).
- Group B: Patients who were candidates for delayed implants ( > 16 weeks after tooth extraction).

Biographic information, main complaints, and medical history were recorded in the patient files. CBCT radiography was prescribed for implant placement.

 Before surgery, a 5-mL non-stimulated salivary sample was collected from patients under aseptic conditions, with no food intake two hours before sampling and mouth rinsing before collection. The samples were then transferred to the medical school’s freezer adjacent to ice packs and stored at -80°C until further testing (measuring ALP enzyme activity) could be performed.

 First, local anesthesia was administered to the target area using 2% lidocaine hydrochloride (Daroopakhsh, Iran). Depending on the implant type, in group A patients, the non-restorable tooth was extracted atraumatically. In group B, a sulcular incision was made in the designated area, and a flap was elevated to provide sufficient visibility and access to the bone. In both groups, drilling of the target area was performed with saline irrigation.

 In the first drilling stage, an implant handpiece with a Lance drill (NSK, South Korea) was used. The implant motor speed was set to 990 rpm clockwise to mark the drilling site on the bone surface. Subsequently, with the Marking drill (Dentis), the area was prepared for implant placement.

 After selecting the appropriate fixture size (diameter and height), the fixture was placed in the prepared site using a handpiece connector (Dentis) with the implant motor speed reduced to 40 rpm. The cover screw (Dentis) was then placed using a screwdriver (Dentis). The flap was returned to its original position and sutured with 3-0 silk thread (SUPA, Iran) using a 19-mm needle to close the soft gingival tissue.

###  Radiography

**Figure 1:**
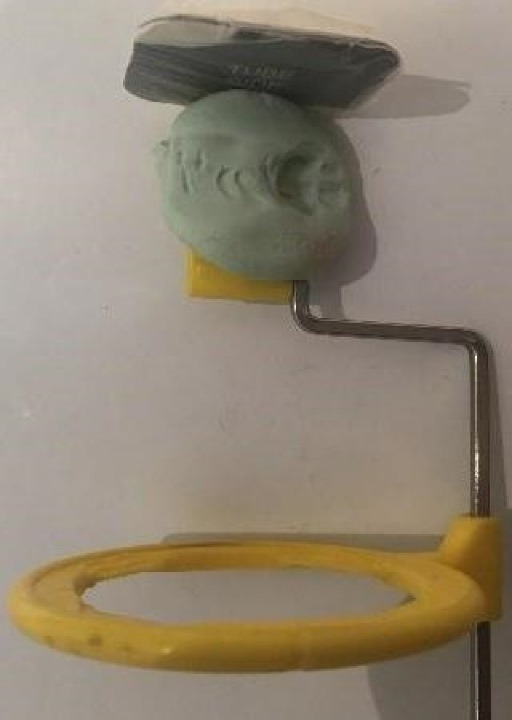
Intraoral radiography with a silicone index

 Immediately after implant placement, a periapical digital intraoral radiograph was taken using the parallel technique with phosphor plate (PSP) sensors. To ensure that all radiographs during follow-up were taken from the same horizontal and vertical angle for comparability, all radiographs were captured using a Posterior XCP and a condensation silicone index. First, the sensor was placed inside the bite block, followed by attaching the alignment rod to the bite block and positioning the aiming ring. The silicone and activator were mixed according to the manufacturer’s instructions and placed in the bite block. The bite block was then positioned in the patient’s mouth, and patients were asked to bite and hold that position for a few seconds until the silicone set. Based on the position of the aiming ring, the horizontal and vertical angle of the x-ray tube was determined. After aligning the x-ray tube, exposure was performed under conditions of 8 mA, 200 ms, and 60 kV (Figure 1).

**Figure 2:**
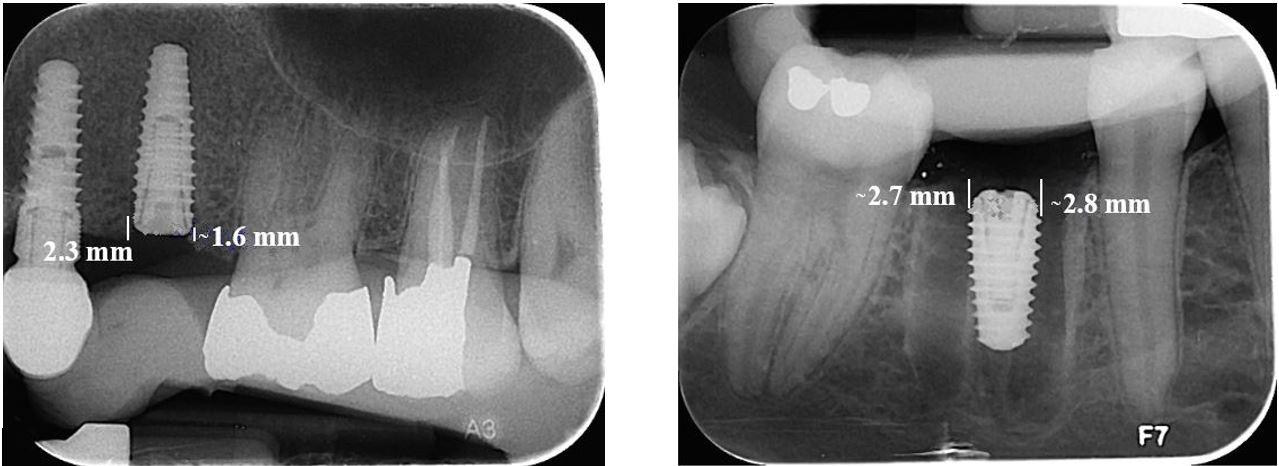
Measuring the height of the crestal bone in the Scanore software

 The intraoral images were analyzed using Scanora software, version 5.2.6. Measurements were taken from both mesial and distal sides of the fixture from the first implant thread to the highest point of crestal bone to assess the alveolar crest level. The measurement from the first radiograph was considered a reference for bone crest height; radiographs taken at two and four months were compared to this reference (Figure 2).

 Postoperative care instructions were given to the patients. Medications were prescribed, including amoxicillin, acetaminophen, ibuprofen, and chlorhexidine mouthwash. The patients were reminded to follow proper oral hygiene practices. Fourteen days after surgery, the patients were scheduled to return for a follow-up appointment. Their sutures were removed, and another 5-mL sample of unstimulated saliva was collected under the previously mentioned conditions.

###  Follow-up sessions

 Two and four months after implantation (before healing abutment placement), a digital periapical radiograph was taken using the same silicone index created during the initial phase. These radiographs were compared with baseline radiographs using Scanora software. In the fourth month, another 5-mL unstimulated salivary sample was collected from patients under previously described conditions.

###  Salivary sample analysis

 To analyze salivary samples, frozen samples were removed from a -80 °C freezer and thawed in an incubator (minimizing time spent outside the freezer). The thawed samples were transferred to Falcon tubes according to predetermined codes and centrifuged in a refrigerated centrifuge at 20 °C for 10 minutes at 5000 rpm.

 Following centrifugation, 1000 microliters of clear supernatant from each sample were separated using a sampler and transferred into coded microtubes for the final analysis of ALP enzyme levels using the Mindray BS-200 device. This device operates based on spectrophotometry, processing samples in a reaction environment without manual intervention; after the incubation period, it provides quantitative results.

## Results

 This study was conducted on 62 implants, with 31 immediate and 31 delayed implants. The age range of participants was 30‒65 years, with a mean age of 46.6 years. In this study, 58% of implants placed in the maxilla were immediate, while this percentage was 41.9% in the mandible.

 As shown in Table 1, salivary ALP activity was higher in immediate implants compared to delayed implants across all the three time points (before surgery, 14 days after surgery, and 4 months after surgery). However, these differences were not statistically significant at any time point.

 According to Table 2, the height of the alveolar crest decreased after the surgical procedure in both immediate and delayed implants. The amount of bone resorption was reported to be greater in immediate implants compared to delayed implants, but the difference was not statistically significant (*P* = 0.06).

 According to Table 3, no significant correlation was observed between salivary ALP levels and the average height of the alveolar crest during follow-up sessions (*P* > 0.05). Pearson’s correlation coefficient also showed negative values, indicating an inverse relationship between ALP activity and alveolar crest height, meaning that as salivary ALP activity increases, alveolar crest resorption occurs.

Table 4 shows salivary ALP activity showed no significant correlation with the studied time intervals (before surgery, 14 days, and 4 months after surgery), gender, or jaw type. However, age significantly correlated with salivary ALP activity, with individuals < 45 years showing lower salivary ALP activity compared to those > 45 years (*P* = 0.04).

 This study also examined the impact of various variables on the mean alveolar crest height. Table 5 shows that over multiple follow-up sessions (immediately after surgery, 2 months, and 4 months afterward), the mean alveolar crest height decreased over time, and this reduction was statistically significant. Gender and the jaw in which the implant was placed had no significant correlation with changes in alveolar crest height. Age was an important factor in these changes. Individuals > 45 exhibited greater reductions in alveolar crest height during the 4-month follow-up compared to those < 45, and this difference was statistically significant.

**Table 1:** Changes in salivary alkaline phosphatase activity at different time intervals in the two studied groups

**ALP**	**Type of Implant**	**Count**	**Mean**	**Standard deviation**	***P*** ** value**
ALP1*	Immediate	31	16.58	10.22	0.44
	Delayed	31	14.45	10.63	
ALP2**	Immediate	31	20.94	15.94	0.84
	Delayed	31	19.52	15.26	
ALP3***	Immediate	31	15.03	10.38	0.93
	Delayed	31	14.81	13.36	

^*^Salivary ALP activity before the surgical procedure. ^**^Salivary ALP activity 14 days after surgery. ^***^Salivary ALP activity 4 months after surgery.

**Table 2:** Changes in crestal bone height in the two study groups

**Type of implant**	**Mean first crest height** ^*^	**Mean secondcrest height** ^**^	**Mean third crest height** ^***^	**Difference between the mean height of the first and second crests**	**Difference between the mean height of the second and third crests**	**Difference between the mean height of the first and third crests**	***P*** ** value**
Immediate	3.85	3.57	3.44	-0.28	-0.12	-0.15	0.06
Delayed	3.83	3.65	3.54	-0.18	-0.10	-0.07	

^*^Mean height of the mesial and distal bone crests immediately after surgery. ^**^Mean height of the mesial and distal bone crests 2 months after surgery. ^***^ Mean height of the mesial and distal bone crests 4 months after surgery.

**Table 3:** Correlation between alkaline phosphatase levels and the average crestal bone height during follow-up sessions

**ALP levels**	Mean first crest height* (Pearson, ***P *** **value)**	Mean second crest height** (Pearson, ***P *** **value)**	Mean third crest height*** (Pearson, ***P*** ** value**
ALP1	0.06, 0.64	0.02, 0.85	0.002, 0.98
ALP2	-0.13, 0.58	-0.11, 0.39	-0.07, 0.29
ALP3	-0.05, 0.65	-0.09, 0.46	-0.10, 0.40

^*^Mean height of the mesial and distal bone crests immediately after surgery. ^**^Mean height of the mesial and distal bone crests 2 months after surgery. ^***^Mean height of the mesial and distal bone crests 4 months after surgery.

**Table 4:** The relationship between alkaline phosphatase activity levels and other variables during follow-up sessions

**Variables**	***P*** ** value**
Time	0.06
Age	0.04
Gender	0.28
Jaw (maxilla, mandible)	0.40

**Table 5:** The relationship between average crestal bone height and other variables during follow-up sessions

**Variables**	***P*** ** value**
Time	0.02
Age	0.01
Gender	0.25
Jaw (maxilla, mandible)	0.97

## Discussion

 One of the most reliable treatment options for replacing missing teeth is implant therapy, which aims to achieve successful prosthetic restoration while restoring proper chewing function and aesthetics.^13,14^ However, like other treatment methods, implant therapy has limitations, and its success is not guaranteed.^15,16^ The success of implant therapy depends on various factors, including oral hygiene, the surgeon’s skill, bone quality, and more. One of the primary long-term success criteria is the stability of the marginal bone around the implant.^16-18^ Different parameters can be used to evaluate marginal bone, with radiographic evaluation being the most effective diagnostic parameter. Biomarkers are also a clinical parameter used for evaluation. ALP is a glycoprotein biomarker attached to the cell membrane that plays a role in bone metabolism.^19-21^ If the increase in ALP is derived from neutrophils, it leads to bone tissue destruction and indicates tissue degradation more rapidly. However, if it originates from osteoblasts, it contributes to bone tissue regeneration.^7,10,11^

 In this study, we observed no significant correlation between salivary ALP levels and changes in alveolar crest height. This aligns with findings by Akhtar et al, who reported similar outcomes regarding the limited reliability of ALP as a biomarker for bone stability in implantology. However, Alshibib and Saleh documented conflicting results, suggesting that higher ALP levels indicate bone regeneration potential around implants. Our findings contrast Alshibib and Saleh’s conclusions, possibly due to differences in the source of salivary ALP, measurement timing, and the follow-up duration. Additionally, studies by Fathima and Harish reported elevated ALP levels in cases of periodontal inflammation, which may suggest that ALP is more reflective of inflammatory processes rather than bone resorption alone. Incorporating these diverse findings, our results suggest that while ALP may indicate inflammatory responses, its role in accurately predicting alveolar bone crest changes remains questionable, particularly in the context of implant stability.^2,7,12^

 In this study, the mean salivary ALP activity in immediate implants was higher than in delayed implants, but the difference between the two groups was not statistically significant. Studies by Fathima and Harish concluded that salivary ALP activity was significantly higher in patients with gingivitis and periodontitis compared to healthy individuals, suggesting that salivary ALP could serve as an inflammatory biomarker.^12^ Considering that tooth extraction is performed simultaneously with the implant procedure in immediate implant surgery, an additional inflammatory process occurs as the tooth socket heals.^22^ Therefore, it would be expected that salivary ALP activity would be significantly higher in immediate implants than in delayed implants. However, this study did not report a significant difference, which may be due to the small sample size or factors influencing tooth socket healing, such as biological variations among individuals or the amount of trauma caused by the surgeon during tooth extraction.^23^

 In this study, mean alveolar crest resorption was higher in immediate implants than in delayed implants; however, the difference was not statistically significant. Studies conducted by Mello et al and Dos Santos Canellas et al also reported no statistically significant difference in alveolar crest resorption between the two types of implants.^24,25^ In contrast, Singh and colleagues’ study concluded that alveolar crest resorption was greater in immediate implants compared to delayed implants. The difference in results between Gagandeep’s and present studies may be due to varying follow-up periods.^5^

 This study found that salivary ALP activity significantly increased with age, while no statistically significant difference in ALP activity was observed based on gender. Recent studies support the connection between salivary ALP levels and age, confirming an increase in ALP activity as individuals approach puberty, followed by a decline after puberty. This pattern aligns with skeletal growth phases where elevated ALP activity corresponds with high bone metabolism during rapid growth phases like adolescence.^26,27^

 Additionally, this study found that individuals > 45 experienced more alveolar crest resorption than those under 45, with overall alveolar crest resorption increasing with age. A study by Negri et al also reported an increase in alveolar crest resorption with age.^28^ However, another study by Al-Fakeh et al. found no statistically significant relationship between age and alveolar crest resorption.^29^ The decrease in bone healing with age can be attributed to reduced stem cell numbers, their proliferation and differentiation potential, and decreased systemic and local blood flow. Thus, increasing age is an important factor contributing to alveolar crest resorption.^1^

 This study also showed no significant relationship between alveolar crest resorption and gender. Al-Fakeh and colleagues’ study reported similar results, which are consistent with our findings.^29^

 There was no statistically significant difference in alveolar crest resorption based on whether the implant was placed in the maxilla or mandible. However, Negri et al reported higher alveolar crest resorption around implants in the maxilla compared to the mandible.^28^ The discrepancy in the results may arise from variations in follow-up durations and the impact of prosthetic forces.

## Conclusion

 The results indicated an association between increased ALP activity and greater alveolar crest resorption. However, salivary ALP is not a reliable diagnostic biomarker for assessing marginal bone condition around implants. Notably, this investigation was conducted over a limited timeframe, which may influence the findings. Future studies with extended follow-up periods are recommended to validate and expand these results.

## Competing Interests

 The authors do not have any financial interests in the companies whose materials were included in this study.

## Consent for Publication

 Not applicable.

## Data Availability Statement

 The data will be shared upon reasonable request by the corresponding author.

## Ethical Approval

 This study was approved by the Ethics Committee of Zanjan University of Medical Sciences, Zanjan, Iran (IR.ZUMS.REC.1401.057).
